# Drug interactions and oncological outcomes: a hidden adversary

**DOI:** 10.3332/ecancer.2019.ed88

**Published:** 2019-03-28

**Authors:** Rachel P Riechelmann, Monika K Krzyzanowska

**Affiliations:** 1 Clinical Oncology Department, AC Camargo Cancer Center, Sao Paulo, Brazil; 2 Division of Medical Oncology and Hematology, Princess Margaret Cancer Centre, Toronto, Ontario, Canada

**Keywords:** drug interactions, cancer, proton-pump inhibitors, therapy

## Abstract

Patients with cancer are at particularly high risk of drug-drug interactions, with approximately 30% of them being exposed to potentially dangerous drug-drug combinations. Yet the real impact of such interactions on oncology practice remains mostly unknown, partly because of the challenges associated with disentangling the effects of harmful interactions from expected side effects of therapy or disease-related symptoms. Recently, some studies have looked at how oncologic outcomes are influenced by drug-drug interactions. In this editorial, we discuss the drug combinations that should be avoided, such as, for example, capecitabine and proton-pump inhibitors, and how research should be conducted in this neglected but clinically relevant topic.

Patients with cancer are at particularly high risk of drug-drug interactions (DDI) because they generally take many drugs during treatment, including cytotoxic and molecularly targeted agents, supportive care medications and drugs to treat comorbidities. Additionally, the pharmacokinetic parameters of many drugs may be altered in cancer patients as a result of impaired absorption secondary to delayed gastric emptying, diarrhea and mucositis, increased volume of distribution resulting from edema, or decreased volume of distribution in patients with sarcopenia-related cancer, decreased drug-protein binding due to hypoalbuminemia and modifed drug metabolism secondary to liver and/or renal dysfunction.

While the synergistic effect between medications may be desired and intended to improve treatment efficacy (e.g. the synergistic effect of leucovorin on 5-fluorouracil), the reality is that most DDI are unintentional and potentially harmful. Previous reports in the literature suggest that 20 to 30% of all adverse reactions to drugs may be caused by interactions between drugs [1]. In studies conducted by our group, nearly one-third of cancer patients receiving cancer-directed therapy or supportive care exclusively [2] were at risk of potential DDI and, when hospitalized, 63% of cancer patients were exposed to at least one drug combination with the potential to interact [3]. Older age, polypharmacy, comorbid conditions, and certain types of cancer such as brain or gynecological tumors were associated with higher risk of potential DDI [2]. Interestingly, the most frequent high-risk combinations involved non-oncological medications, such as steroids, phenytoin, and warfarin [4], although few oral anticancer drugs were in use at the time of these studies. And oral cancer-directed therapies might be particularly prone to DDI. However, the real impact of DDI on oncology practice including on patient outcomes and healthcare costs remains mostly unknown partly because of the challenges associated with disentangling the effects of harmful interactions from the expected side effects of therapy or disease related symptoms.

Because it is difficult to determine causality, few studies have evaluated real DDI in oncology. In a retrospective study conducted by our group, we found that 2% of unplanned hospital admissions among 298 cancer patients were caused by a real DDI [5]. Examples of DDI that led to hospitalization were phenytoin and warfarin (deep venous thrombosis due to warfarin induced hepatic metabolism by phenytoin), captoril and dexamethasone (severe high blood pressure), warfarin plus omeprazole (upper digestive bleeding) and aspirin plus enoxaparin (severe melena) [5].

More recently, studies have raised the very important question of whether certain DDI influence, and to what extent, the oncological outcomes of patients with cancer. A secondary analysis of the phase III trial TRIO-013/LOGiC, which randomized HER2-positive metastatic gastric patients to receive standard chemotherapy with or without lapatinib, showed that concomitant use of gastric acid suppressants, such as proton pump inhibitors (PPIs), with capecitabine was associated with lower antitumor efficacy of capecitabine. PPI-treated patients had lower median progression-free survival (4.2 vs 5.7 months; HR 1.55; p < .001), median overall-survival (9.2 vs 11.3 months HR 1.34; p = .04) and disease control rate (72% vs 83%; p = .02) compared with patients who were not taking PPIs during the trial. The explanation for these findings is a pharmacokinetic DDI, where substantial reduction in gastric acidity impairs the dissolution of capecitabine which in turn lowers absorption of the drug into the systemic circulation [6]. Similarly, a retrospective study of 389 patients with stage II-III colorectal cancer who received adjuvant CapeOx or FOLFOX demonstrated that PPI use during chemotherapy negatively affected the recurrence-free survival in CapeOx-treated patients. In the subgroup of CapeOx, the 3-year recurrence-free survival rate was significantly lower among PPI users: 69.5 *versus* 82.6% (p = .029; Hazard Ratio [HR] = 2.03; 95% Confidence Interval 1.06-3.88; p = .033). PPI use did not influence the outcomes of FOLFOX-treated patients [7]. Because CapeOx use may increase following the publication of the IDEA study [8], which demonstrated the non-inferiority of 4 cycles of CapeOx to 12 cycles of FOLFOX in low risk stage III colon cancer, the issue of interaction between capecitabine and PPIs becomes extremely relevant. The absolute difference in 3-year RFS rate of 13% depending on PPI use is certainly of considerable magnitude, potentially compromising the outcomes in some patients. In fact, the absolute difference in the relapse free survival rate based on concomitant PPI use is greater than the benefit seen from the addition of oxaliplatin to a fluoroupyrimidine in the adjuvant setting. The use of PPI in association with pazopanib was also demonstrated to be detrimental. In a pooled analysis of clinical trials of pazopanib in soft tissue sarcoma, 333 patients who took a gastric acid-reducing agent for at least 80% of treatment duration experienced inferior median progression-free survival (HR: 1.49; 2.8 vs 4.6 months, p = 0.01), shorter median overall survival (HR: 1.81; 8 vs 12.6 months, p < 0.01) [9]. Through a similar pharmacokinetic mechanism, the concomitant use of acid-reducing agents impairs the absortion of erlotinib [10]. While the clinical impact of combining a PPI with erlotinib has not been evaluated in terms of oncological outcomes, this drug combination should be avoided.

Another important mechanism of pharmacokinetic DDI is the interference, through either induction or inhibition, with the activity of the cytochrome P450 (CYP) system in the liver; the isoenzymes CYP3A4, CYP1A2, CYP2C9 and *CYP2D6* are responsible for more than 90% of drug oxidation in humans [11, 12]. A specific example of such a DDI is the combination of tamoxifen and selective serotonin reuptake inhibitors (SSRI), often used to treat depression or vasomotor symptoms induced by tamoxifen [13]. In a prospective study, the SSRI paroxetine led to a 58 to 64% decrease in the plasma concentration of endoxifen (an active tamoxifen metabolite) in women with wild-type CYP2D6 genotype and by 24% in women with a non-functional *CYP2D6* genotype [14]. These data indicate that plasma concentrations of tamoxifen metabolites might be directly affected by polymorphisms of *CYP2D6* as well as by SSRIs. However, no differences in toxicity, breast cancer recurrence or mortality were observed in a study that evaluated women receiving SSRIs that inhibit CYP2D6 (paroxetine and fluoxetine) or SSRIs that do not inhibit CYP2D6 (citalopram, escitalopram, fluvoxamine and sertraline) over 16,000 women followed up to 14 years [15]. Since *CYP2D6* polymorphisms vary across people, results from these studies [14, 15] may not be generalizaled to other populations. While definitive recommendations about the use of SSRI and tamoxifen cannot be made [13], we think such combinations, particularly tamoxifen and SSRI that inhibit CYP2D6, should be avoided, if possible; if not, the uncertainty involving such drug combination should be presented to patients for an informed decision to be made.

The above examples are just a few of the known adverse effects of DDI. While the real impact of DDI on oncologic outcomes is unknown, there is enough evidence to suggest that they can lead to unnecessary hospitalizations, high healthcare costs and may negatively impact cancer outcomes. As such, there is an imperative at the institutional and provider levels to prevent deleterious DDI in cancer patients. Institutions should invest in screening software that provides electronic alerts for dangerous DDI to health professionals and establish clinical pharmacist teams to help with medication reconcilliation and DDI evaluation. Physicians should be familiar with unsafe drug combinations used in their field of expertise, and be particularly attentive to DDI among patients at greater risk such as those with multiple comorbidities or on many medications or on high risk medications such as warfarin or anticonvulsants. Healthcare professionals should also consider whether DDI may have contributed to unexpected adverse events.

On a brighter side, studies should also explore the potential positive side of drug-drug or drug-food interactions, where an interacting combination is intentionally prescribed to improve outomes or decrease costs. For example, a randomized phase II trial in men with hormone-sensitive prostate cancer showed that low-dose abiraterone acetate (LOW 250 mg) administered with a low-fat meal offered similar efficacy in terms of PSA response when compared with standard dose abiraterone (STD 1,000 mg) taken while fasting [16]. Such studies may help facilitate the incorporation of expensive medications in low-resource countries, thereby improving access to effective therapies in these settings.

## Conclusion

In conclusion, drug combinations with potential to interact are common, yet the impact of these interactions on the ouctomes of patients with cancer remains largely undetermined. Collaborative efforts should be undertaken among academic institutions to conduct high quality studies on DDI, with particular focus on cancer-related outcomes, healthcare costs and to identify currently unknown but clinically meaningful DDI.

## Conflicts of interest

The authors have no conflicts of interest to declare.

## Funding statement

The authors did not receive any funding for this editorial.
